# Supplementary material for: Discovery of ACE Inhibitory Peptides Derived from Green Coffee Using In Silico and In Vitro Methods
Source: Foods. 2023 Sep 19;12(18):3480. doi: 10.3390/foods12183480 (PMC10529643; doi:10.3390/foods12183480)
Supplement: Supplementary file 1 [file foods-12-03480-s001.zip › foods-2565959-supplementary.pdf]

Supplementary Materials for

Discovery of ACE Inhibitory Peptides Derived from Green Coffee Using *in silico* and *in vitro* Methods

Hao-Peng Dai[1,2], Min He[1,2], Gui-Lin Hu[1,2], Zhong-Rong Li[1], Abdulbaset Al-Romaima[1,2], Zhou-Wei Wu[1,2], Ming-Hua Qiu[* 1,2]

[1] State Key Laboratory of Phytochemistry and Plant Resources in West China, Kunming Institute of Botany, Chinese Academy of Sciences, Kunming 650201, People's Republic of China

[2] University of Chinese Academy of Sciences, Beijing 100049, People's Republic of China

* Corresponding author:

Telephone: +86-0781-65223327. Fax: +86-0871-65223325.

E-mail: mhchiu@mail.kib.ac.cn

Table S1 Peptide sequence, retention time (RT), length, molecular mass, charge and score of the peptides identified in the alcalase hydrolysates using nanoLC-MS/MS.

No.	Peptide sequence	RT (min)	length	Mass (Da)	charge	score
1.	VVINPGNPTGQ	8.4084	11	1094.570861	2	186.4107611
2.	GLPSGGAPSGY	8.4838	11	961.452821	2	185.0500457
3.	NLGSIPTQ	9.0844	8	828.4363092	2	125.8140196
4.	GLPASPGAAVGQ	9.1765	12	1023.53823	2	128.3831953
5.	KNPNIPDPNTLM	9.3902	12	1368.681202	2	102.0614523
6.	FEDNAGVIVNPK	9.7198	12	1301.665422	2	157.6630823
7.	SENIGLPQ	10.83	8	856.4290199	2	189.5286939
8.	TVDKRLL	12.363	7	843.5099303	2	102.9798471
9.	GESFWGGQ	14.831	8	866.3576558	2	152.389641
10.	ALDPGLTY	14.954	8	848.4254938	2	122.1542868
11.	RVDSIPIL	15.156	8	911.5414324	2	100.0663334
12.	TNDNAMINPL	18.482	10	1101.512101	2	166.5163046
13.	ILMIGTQ	19.225	7	774.42865	2	117.561596

No.	Peptide sequence	RT (min)	length	Mass (Da)	charge	score
14.	LILGPDSPAVQ	19.243	11	1108.613483	2	93.26691455
15.	GQLIIVPQ	21.175	8	866.5232251	2	119.3001685
16.	SDVGLERQ	23.314	8	902.4490947	2	103.8329972
17.	VIEGDLL	24.948	7	757.4245332	1	97.64023512
18.	METSNSVPSIL	25.752	11	1192.557553	2	61.96230632
19.	ALATPLL	26.365	7	697.4379556	1	96.33678951
20.	IPLDLNY	27.34	7	846.4484963	1	71.35928969
21.	IIPNEVY	27.745	7	846.4484963	1	67.03480014
22.	MSQAGVFDVGY	29.401	11	1214.533312	2	55.53053424
23.	AGPGGWNDPDML	31.269	12	1228.52441	2	103.8329972
24.	GGKADVL	32.044	7	658.3662592	1	66.99353222
25.	STSATVVGMAVSQ	32.974	13	1252.596803	2	57.59840786
26.	VGAGNIPPYSPM	35.197	12	1217.583589	2	59.24507599
27.	DIIEFIQ	36.02	7	876.4603733	1	69.99846331
28.	DAKMAGM	36.844	7	754.3000558	2	56.51017139

No.	Peptide sequence	RT (min)	length	Mass (Da)	charge	score
29.	KNPNIPDPNTL	38.286	11	1221.634336	2	105.0319149
30.	VADPDKLPTIPGQ	38.293	13	1349.722628	2	125.9702909
31.	VASGNVL	38.575	7	658.3658152	1	98.03023713
32.	TNEILIGK	39.776	8	886.5099528	2	114.4976386
33.	ALRALPE	40.287	7	768.4482693	1	111.2783061
34.	SALRAIPE	41.283	8	855.4807962	2	101.5418696
35.	ADLSRIDL	41.69	8	901.4867068	2	125.4976377
36.	ADSLDLRL	42.098	8	901.4867068	2	107.4293946
37.	GDAPRVL	42.131	7	726.4051323	2	98.4148342
38.	APIAVGDVIPDGTL	42.156	14	1336.723736	2	120.8684288
39.	SPVAPLAPVTL	42.264	11	1063.629482	2	155.8763914
40.	AATLPLM	44.351	7	715.3962087	1	61.3249463
41.	ILLPGFTQ	45.431	8	887.5116097	2	187.6333307
42.	IPAGMLL	45.697	7	713.415722	1	58.16054904
43.	AFNVDLK	46.161	7	805.4360244	2	129.3622389

No.	Peptide sequence	RT (min)	length	Mass (Da)	charge	score
44.	SAFRAIPE	46.285	8	889.4672821	2	124.080105
45.	GYIPGIIY	46.852	8	894.4857466	2	100.9062997
46.	EGDGGVGTIKL	48.117	11	1044.542237	2	150.6187803
47.	IQIIFPE	48.639	7	858.4837469	2	120.8974524
48.	GIESVPAALIGL	49.008	12	1138.657976	2	138.8255169
49.	APILDEVAVSL	49.664	11	1125.621915	2	113.4992147
50.	AKDPVRVL	50.347	8	938.5562757	2	105.1022565
51.	RAIPEEVL	51.483	8	925.5223414	2	134.6632081
52.	SAERGFLY	52.378	8	941.4648164	2	133.5998691
53.	GVKSVEIL	52.454	8	843.5052991	2	116.5422743
54.	AITPPVMLPPL	52.876	11	1147.664709	2	94.66240868
55.	VGHTDTARMLL	58.578	11	1228.628283	2	63.56507644

Table S2 Peptide sequence, retention time (RT), length, molecular mass, charge and score of the peptides identified in the thermolysin hydrolysates using nanoLC-MS/MS.

No.	Peptide sequence	RT (min)	length	Mass (Da)	charge	score
1.	LLERGPTPEP	6.6184	10	1107.581739	2	98.2328978
2.	AVNHPNFPST	6.976	10	1082.504788	2	117.8851479
3.	FWDSNNPE	13.303	8	1007.39407	2	140.981477
4.	FVDPDGWKT	13.345	9	1063.493388	2	141.5204132
5.	FLPEYSEQ	15.048	8	1011.447063	2	128.5660771
6.	VKNPNPIPIP	18.395	10	1087.629915	2	104.3656055
7.	LFPSPSPPPP	18.679	10	1034.533909	2	128.5660771
8.	VLETPDGPL	19.805	9	939.482591	2	148.2059973
9.	FDDEVKQGQL	22.75	10	1177.551339	1	72.64306786
10.	LITMEPNSL	23.211	9	1016.516528	2	175.9971999
11.	IGLPQEAD	23.286	8	841.4118628	2	128.1929192
12.	ITPPVMLPP	29.942	9	963.5385208	2	163.3414491
13.	IFDPFPSD	31.76	8	936.4138101	2	163.9965319

No.	Peptide sequence	RT (min)	length	Mass (Da)	charge	score
14.	FRFPSEAG	33.532	8	909.4325214	1	66.99316637
15.	FHPPGSDRVD	36.265	10	1125.515555	3	81.29628213
16.	IDWKETPEAH	36.317	10	1224.565717	2	78.93422344
17.	VVGDPLDPNSHHGPQ	37.898	15	1567.731524	3	99.75195723
18.	VMKNRPISEE	38.434	10	1217.608274	2	107.0075092
19.	VMDDTSESKPQHPSR	39.684	15	1712.762287	4	80.5339083
20.	IDWKETPEAHV	41.274	11	1323.633694	3	90.05030949
21.	YSPDGEEGFPGNL	58.488	13	1380.574942	2	88.13613616
